# The complete chloroplast genome of *Keteleeria evelyniana* Mast var. *pendula* Hsüeh (Pinaceae), a species with extremely small populations in China

**DOI:** 10.1080/23802359.2024.2345780

**Published:** 2024-04-26

**Authors:** Guan-Song Yang, Yu Qiu, Zheng-An Yang

**Affiliations:** College of Horticulture and Landscape, Yunnan Agricultural University, Kunming, China

**Keywords:** Chloroplast genome, *Keteleeria evelyniana* var. *pendula*, phylogeny

## Abstract

*Keteleeria evelyniana* Mast var. *pendula* Hsüeh, a typical plant species of extremely small population, is faced to be endangered. The complete chloroplast (cp) genome of *K. evelyniana* var. *pendula* has been assembled and annotated for the first time in this study. The complete genome in length was found to be 117,139 bp. The genome annotation revealed a total of 118 genes, including 34 transfer RNA (tRNA) genes, 4 ribosomal RNA (rRNA) genes, and 80 protein-coding genes. The maximum-likelihood phylogenetic tree supported that *K. evelyniana* var. *pendula*, *K. fortune*, *K. evelyniana*, and *K. davidiana* are clustered in one branch. This complete chloroplast genome helped us to understand the evolution of *K. evelyniana* var. *pendula*. These results laid the foundation for future studies on the conservation of this species.

## Introduction

Coniferous forests, which are very important to ecosystems and human society, have been the dominant type of forest for more than 200 million years (Nystedt et al. 2013). Pinaceae is an important family in coniferous forests, with 10 genera (including *Abies*, *Keteleeria*, *Pseudotsuga*, *Tsuga*, *Picea*, *Cathaya*, *Larix*, *Pseudolarix*, *Cedrus*, and *Pinus*) and about 35 species distributed in China (Zhengyi et al. 2005, 2008). *K. evelyniana* var. *pendula* (i.e. a variety of *K. evelyniana* discovered by Ji-ru Xue in 1983) is a perennial woody plant of the genus *Keteleeria* in the family Pinaceae (Xue 1983). It is distributed narrowly in Huaning County, Yunnan Province, China (Jiang et al. 2008; Li J, et al. 2021). The high ornamental value of this species due to its unique shape has led to severe damage to wild resources and it is now an endangered species, listed as a second-class national key protected wild plant and IUCN Red List in China (Walter and Gillett 1998; Xie et al. 2017). In addition, this species was listed as one of the plant species of extremely small population and requires urgent rescue action (Sun 2013). Evolutionary and phylogenetic explorations are essential for species conservation, while the species is poorly understood (Yang et al. 2017, 2020). To address the urgent need for conservation efforts, it is crucial to gain a comprehensive understanding of the genetic characteristics and evolutionary history of this species. Therefore, the complete chloroplast genome of *K. evelyniana* var. *pendula* was reported in this study. The results lay the foundation for further conservation of this endangered species.

To date, chloroplast genome has been widely used to analyze the phylogenetic and domestication of higher plants (Nie et al. 2020). Chloroplast genome sequence has also been shown to have the potential for understanding structural and functional evolution (Sabater 2018). Chloroplast genomes have been reported for a number of species in the genus *Keteleeria*, such as *K. davidiana* var. *calcarea* and *K. evelyniana* (Li GY, et al. 2019; Li JJ, et al. 2021). However, there are few reports on the chloroplast genome of *K. evelyniana* var. *pendula*. This species is vital for understanding the evolutionary relationship of the genus *Keteleeria*.

This study aims to provide a comprehensive chloroplast genome sequence for future genetic and phylogenetic studies. To investigate the evolutionary relationships of *K. evelyniana* var. *pendula* within the genus *Keteleeria*, we constructed a phylogenetic tree based on the complete chloroplast genome.

## Materials and methods

**Figure 1.**
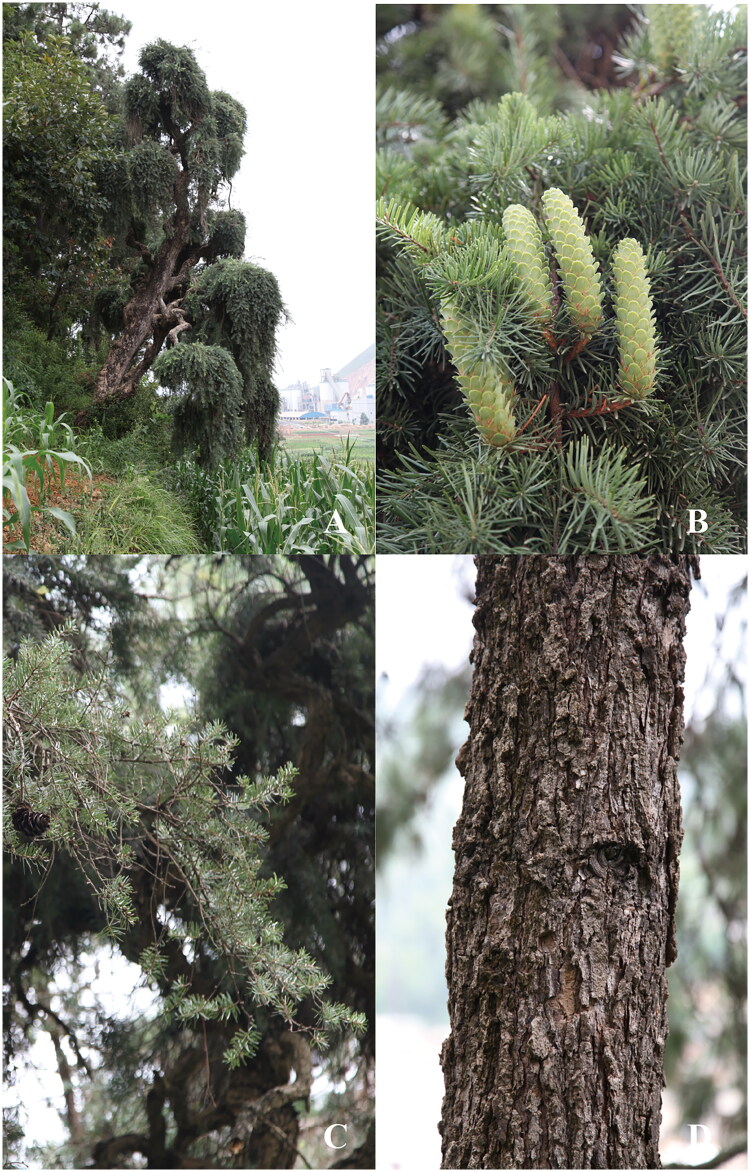
*K. evelyniana* var. *pendula* distributed in Huaning County, Yuxi City, Yunnan, China (these photographs were taken by Guan-Song Yang). *K. evelyniana* var. *pendula* is an evergreen tree. The main stem is twisted and branched; the bark is relatively hard; the branches are long and drooping. The leaves are needle-shaped, arranged in two rows on the lateral branches, with a blunt tip that is often slightly raised at the apex and a wedge-shaped base, the front side is shiny green. The cones are initially upright, but droop when mature, the apex of the cone scales is noticeably outwardly curved, with a slight rust-colored hair on the back, the bract scales have three lobes at the apex, with rounded tips on both sides and a smaller and sunken Central lobe, the upper part of the winged seeds is wider. (A) Plant panorama of *K. evelyniana* var. *pendula*, (B) Cones of *K. evelyniana* var. *pendula*, (C) Leaves of *K. evelyniana* var. *pendula*, (D) Tree trunk of *K. evelyniana* var. *pendula.*

Research materials were collected from the Plaza of Huaning County, Yuxi City (with longitude of 102.9393 and latitude of 24.1883) (Figure 1). The plant material was used with permission from Yunnan Agricultural University and Yuxi Forestry Bureau. The voucher specimens were deposited in the College of Horticulture and Landscape of Yunnan Agricultural University with the voucher number: YAU-20210207 (contact: Guan-Song Yang, gsyang105@163.com). The total DNA was extracted using the CTAB method (Doyle 1991). The chloroplast whole genome DNA data of this species was sequenced using the Illumina NovaSeq 6000 platform (Quail et al. 2008). It was assembled using the GetOrganelle program (Jin et al. 2020). The assembled chloroplast genome was annotated by the combination of PGA (Plastid Genome Annotator) (Qu et al. 2019) and GeSeq (accurate annotation of organelle genomes) (Tillich et al. 2017). In this work, the chloroplast genome annotation was completed with *K. davidiana* (MW580774.1) as the reference. PGA and GeSeq methods were adopted to improve the accuracy of the annotation (Liu et al. 2012). During this process, differential genes were checked, incorrect annotations and redundant annotations were removed, and multiple exon boundaries were identified to obtain the final annotation. The Genome map can be carried out using CPGView (http://www.1kmpg.cn/cpgview). Phylogenetic analysis was carried out using 21 published species within the Pinaceae and one outgroup (*Cathaya argyrophylla*) to confirm the phylogenetic position of *K. evelyniana* var. *pendula* (Supplementary Table 1). These sequences were aligned using MAFFT (v7.450) (Rozewicki et al. 2019). Maximum-likelihood (ML) analysis of tree topologies based on mutually consistent GTR models was carried out with strong support for all branches in the system tree using RA × ML 8.0 software (Stamatakis 2014).

## Results

**Figure 2.**
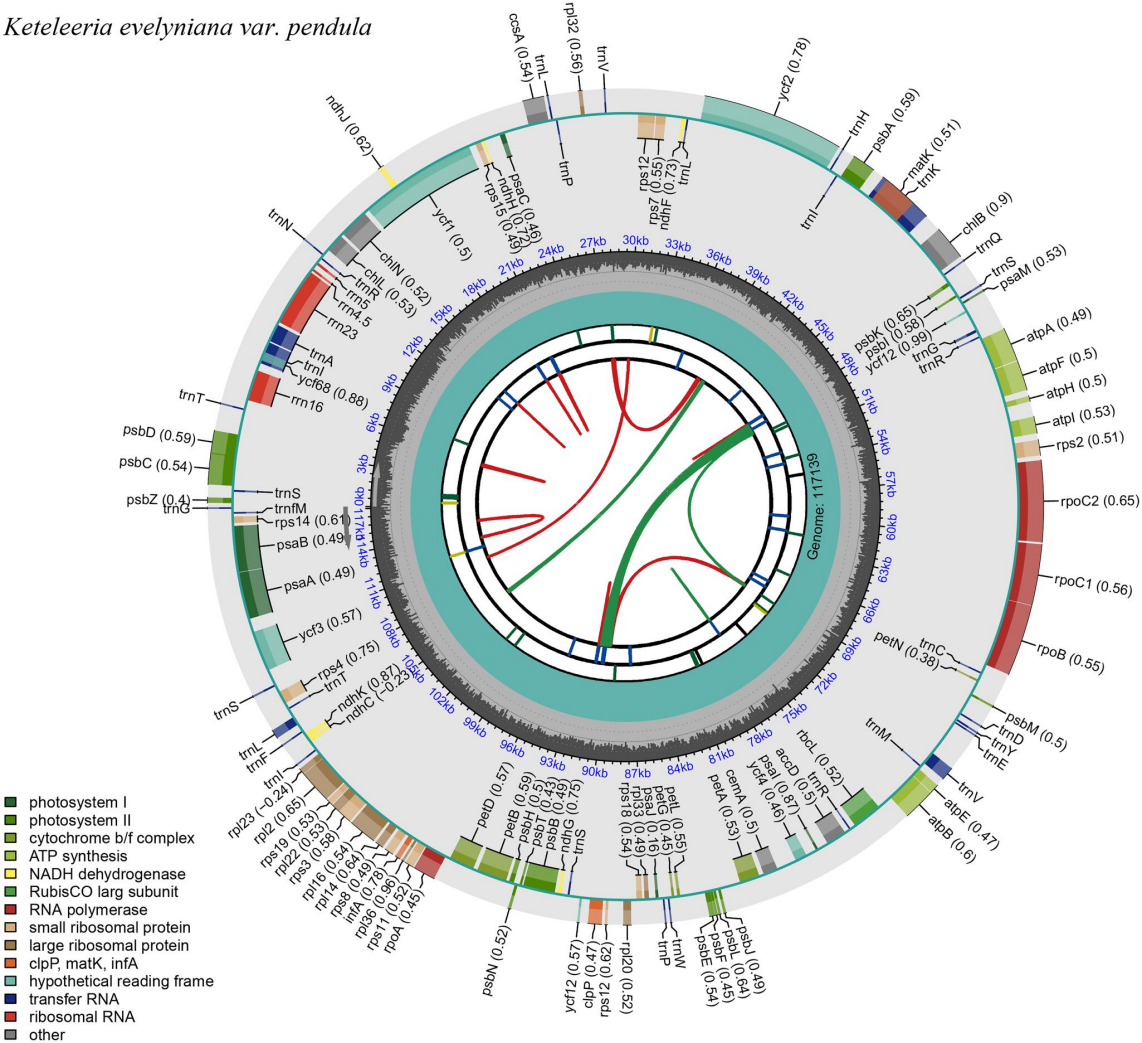
The chloroplast genome map of *K. evelyniana* var. *pendula* shows the regions of 118 genes. The inner circle of darker gray color represents the guanine and cytosine (GC) content, and of lighter gray color represents the adenine and thymine (at) content of the chloroplast genome. The genes drawn outside and inside of the outer circle are transcribed counterclockwise and clockwise. This map can be carried out using CPGView (http://www.1kmpg.cn/cpgview).

**Figure 3.**
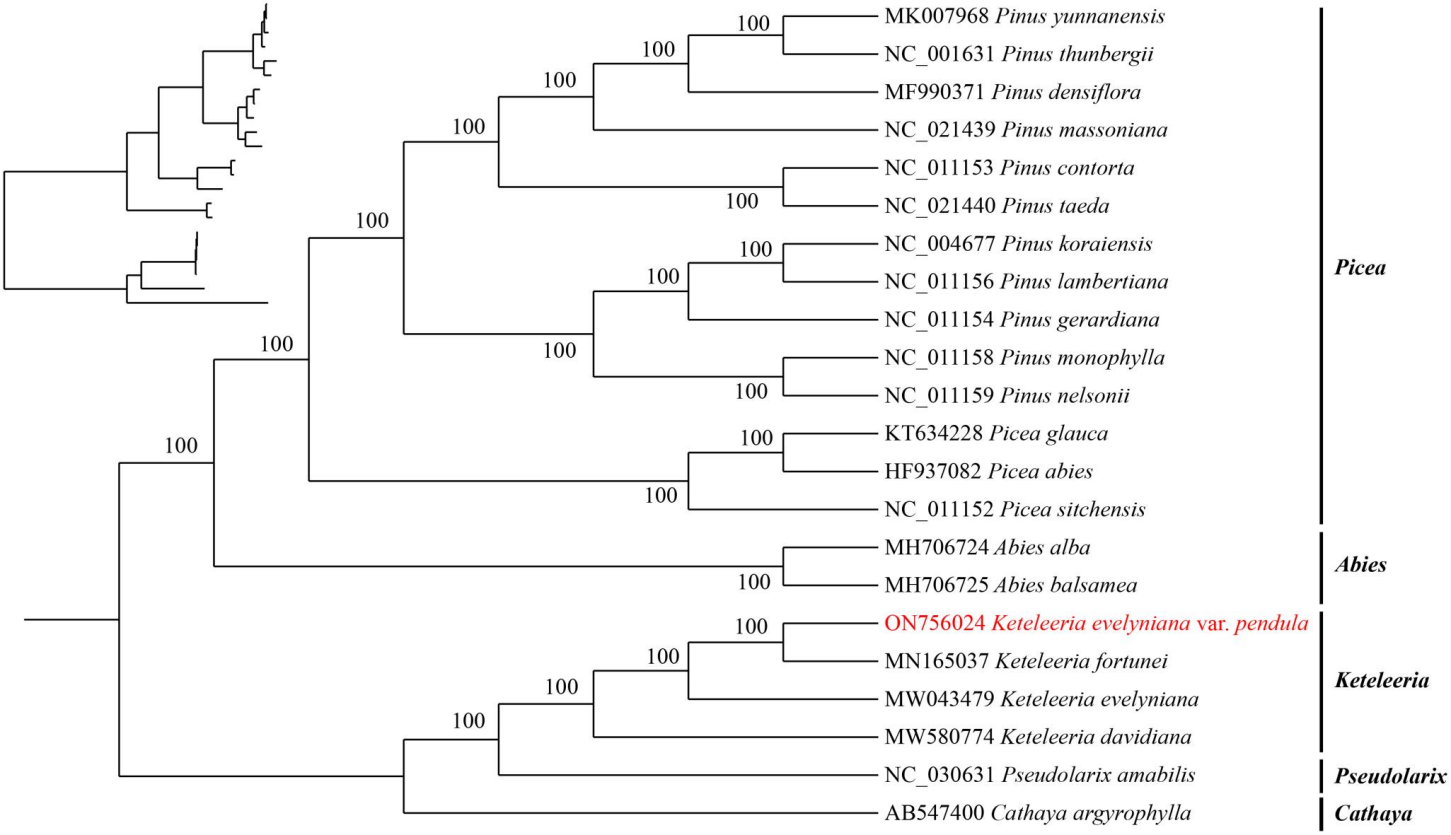
Maximum-likelihood phylogenetic tree using 21 published species within the Pinaceae and one outgroup (*Cathaya argyrophylla*). The phylogenetic tree was constructed using the maximum-likelihood method (ML) and bootstrap was performed 1000 times. The following sequences were used: *K. evelyniana* var. *pendula* ON756024, *K. fortune* MN165037 (Li HT, et al. 2019), *K. evelyniana* MW043479 (Li JJ, et al. 2021), *K. davidiana* MW580774 (Zhang et al. 2021) *Abies Alba* MH706724 (Li GY, et al. 2019), *A. balsamea* MH706725, *Pseudolarix amabilis* NC030631 (Sudianto et al. 2016), *Pinus densiflara* MF990371 (Kim et al. 2018), *P. yunnanensis* MK007968 (Hong et al. 2020), *P. lambertiana* NC011156 (Cronn et al. 2008), *P. koraiensis* NC004677, *P. gerardiana* NC011154 (Cronn et al. 2008), *P. nelsonii* NC011159 (Cronn et al. 2008), *P. monophylla* NC011158 (Cronn et al. 2008), *P. contorta* NC011153 (Cronn et al. 2008), *Pinus taeda* NC021440, *P. thunbergii* NC001631, *P. massoniana* NC021439 *Picea abies* HF937082, *P. glauca* KT634228 (Parmar et al. 2022), *P. sitchensis* NC011152 (Cronn et al. 2008), *Cathaya argyophylla* AB547400 (Lin et al. 2010). The sequences used for the tree structure are coding sequences, and the bootstrap support values are shown on the nodes.

The chloroplast genome of *K. evelyniana* var. *pendula* in length was found to be 117,139 bp (Figure 2), average reference genome coverage of 99.57%, and average sequencing depth of 99 × (Supplementary Figure S1). It is not a typical quadruplex structure and does not consist of repeat region A (IRA) and repeat region B (IRB) (Figure 2). The genome structure is similar to that of Leguminosae and Algae (Kim and Cullis 2017; Zhu et al. 2019). A circular map of the chloroplast genome and a schematic map of the *cis* splicing genes and *trans* splicing genes (Supplementary Figure S2) were visualized by CPGView. The chloroplast genome contained a total of 118 genes, including 34 tRNA genes, 4 rRNA (rrn16, rrn23, rrn4.5, rrn5) genes, and 80 mRNA genes (Supplementary Table 2). The chloroplast genome of *K. evelyniana* var. *pendula* is structurally similar to that of other Pinaceae species, such as the genome size, structure, expansion, and contraction of inverted repeat (IR) boundaries. The total GC content of *K. evelyniana* var. *pendula* chloroplast genome reaches 38.52%, similar to that of other members of the Pinaceae and angiosperms in general (Li GY, et al. 2019; Li JJ, et al. 2021). This suggested that the length, characteristics, and genes in the chloroplast genome of *K. evelyniana* var. *pendula* differed from *K. evelyniana*. The annotated chloroplast genome has been submitted to GenBank (under the accession number ON756024.1). The raw sequencing reads used in this study were deposited in the public repository SRA under the accession number SRR19737605. The ML phylogenetic tree supported that *K. evelyniana* var. *pendula*, *K. fortune*, *K. evelyniana*, and *K. davidiana* are clustered in one branch. The *K. evelyniana* var. *pendula* and *K. fortune* are more closely related clusters in the genus *Keteleeria* (Figure 3). This is consistent with previous studies on Pinaceae (Li GY, et al. 2019). The *K. evelyniana* var. *pendula* chloroplast genome would provide a solid foundation for phylogenetic and evolutionary studies in *Keteleeria*. The results can facilitate the development of conservation strategies for *K. evelyniana* var. *pendula*.

## Discussion and conclusion

In this study, the structure of this species was annotated and the chloroplast genome of *K. evelyniana* var. *pendula* was sequenced for the first time. Furthermore, the structure of this species has been meticulously annotated, shedding light on its unique characteristics. The genome structure is similar to that of Leguminosae and Algae (Kim and Cullis 2017; Zhu et al. 2019). The successful assembly of the chloroplast genome sequence of *K. evelyniana* var. *pendula* and the comprehensive annotation of its structure represent important milestones in our understanding of this species. The phylogenetic analysis conducted as part of this study has yielded intriguing results. The ML phylogenetic tree supported that *K. evelyniana* var. *pendula*, *K. fortune*, *K. evelyniana*, and *K. davidiana* are clustered in one branch. The *K. evelyniana* var. *pendula* and *K. fortune* are more closely related clusters in genus *Keteleeria*. These results provide new insights into the phylogenetic relationships of the family and are consistent with previous studies on Pinaceae (Li Y, et al. 2019). This finding has significant implications for our understanding of the phylogenetic relationship within the Pinaceae family. By providing new and valuable information on the phylogenetic relationship of the Pinaceae family, this study contributes to the growing body of knowledge in the field. Besides, this study will complement the chloroplast genomic data collected in China for *Keteleeria*, which is of great significance for the investigation and conservation of germplasm resources of this genus.

The chloroplast genome of *K. evelyniana* var. *pendula* is 117,139 bp in length. It contains 118 genes, with a GC content of 38.52%. Phylogenetic analysis shows *K. evelyniana* var. *pendula*, *K. fortune*, *K. evelyniana*, and *K. davidiana* clustered together. This genome provides a basis for phylogenetic and evolutionary studies, while also providing a scientific basis for the conservation of this species.

## Supplementary Material

Supplemental Material

Supplemental Material

Supplemental Material

## Data Availability

The *Keteleeria evelyniana* var. *pendula* genome sequence data are available in GenBank under accession number ON756024.1 (https://www.ncbi.nlm.nih.gov/nuccore/2295471757). The associated BioProject, SRA, and Bio-Sample numbers are PRJNA848921, SRR19737605 (https://www.ncbi.nlm.nih.gov/sra/?term=SRX15783530), and SAMN29019768, respectively.
