# Supplementary material for: The complete chloroplast genome of Keteleeria evelyniana Mast var. pendula Hsüeh (Pinaceae), a species with extremely small populations in China
Source: Mitochondrial DNA B Resour. 2024 Apr 26;9(4):557–62. doi: 10.1080/23802359.2024.2345780 (PMC11057466; doi:10.1080/23802359.2024.2345780)
Supplement: Supplemental Material [file TMDN_A_2345780_SM4383.pdf]

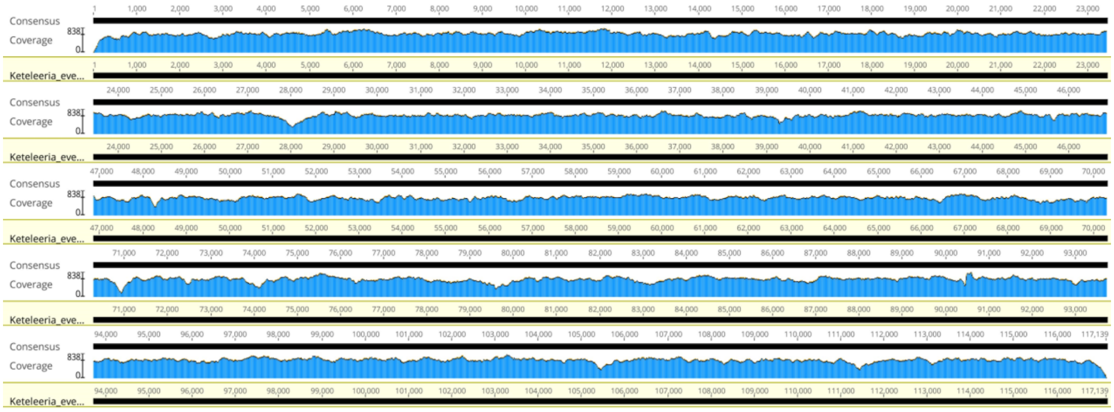

Supplementary Figure S1. The coverage plot across the assembled chloroplast genome of *Keteleeria evelyniana* var *pendula*.

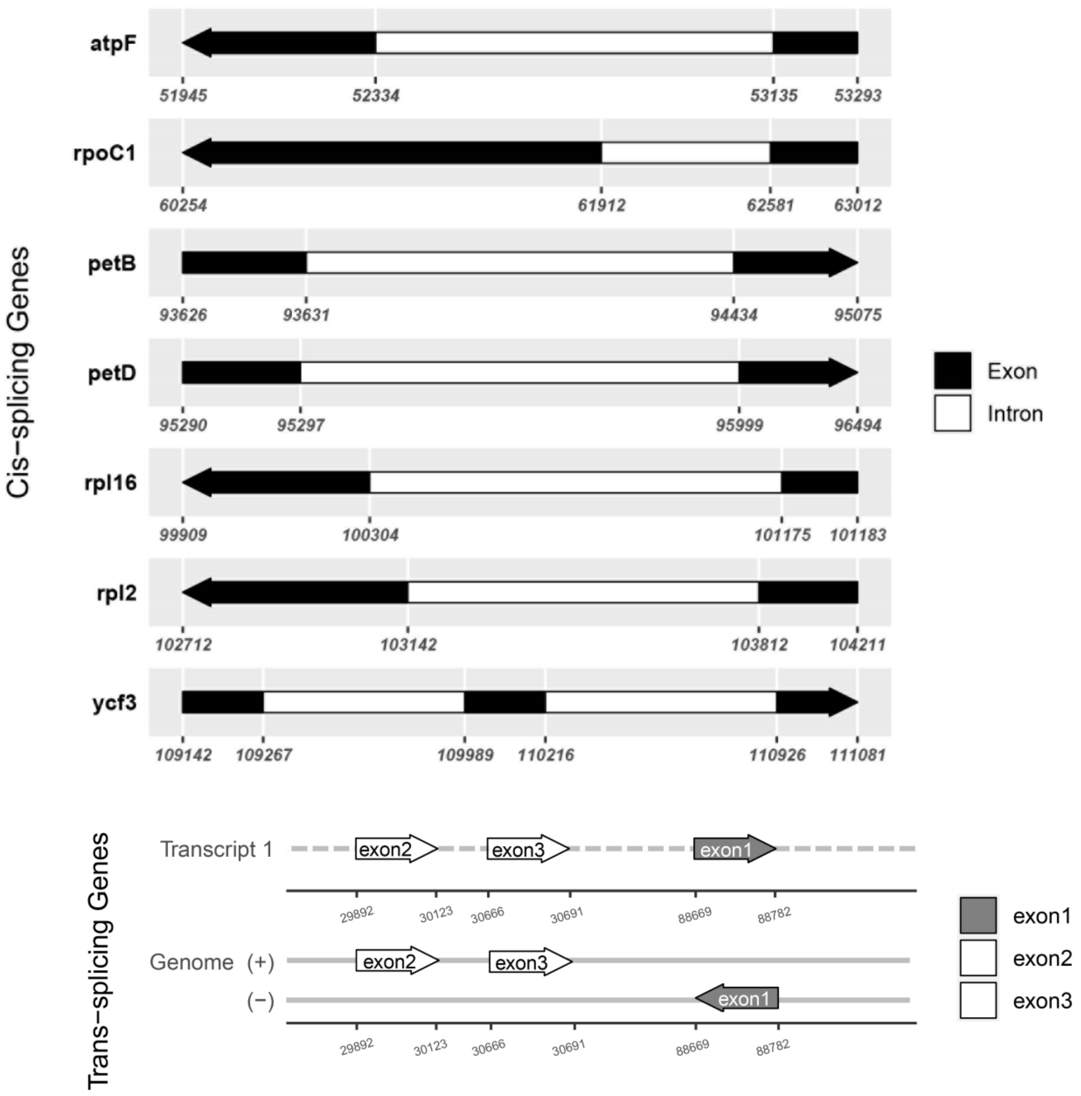

Supplementary Figure S2. Schematic map of the cis-splicing and trans-splicing genes. A, cis-splicing genes; B, trans-splicing genes.
