# Supplementary material for: The complete chloroplast genome of Keteleeria evelyniana Mast var. pendula Hsüeh (Pinaceae), a species with extremely small populations in China
Source: Mitochondrial DNA B Resour. 2024 Apr 26;9(4):557–62. doi: 10.1080/23802359.2024.2345780 (PMC11057466; doi:10.1080/23802359.2024.2345780)
Supplement: Supplemental Material [file TMDN_A_2345780_SM4364.pdf]

Supplemental Table 2 Chloroplast gene classification statistical table

Category	Gene group	Gene name
Photosynthesis	Subunits of photosystem I	*psa*A,*psa*B,*psa*C,*psa*I,*psa*J,*psa*M
	Subunits of photosystem II	*psb*A,*psb*B,*psb*C,*psb*D,*psb*E,*psb*F,*psbH*,*psb*I,*psbJ*,*psb*K,*psb*L,*psb*M,*psb*N,*psb*T,*psb*Z
	Subunits of NADH dehydrogenase	*ndh*C,*ndh*F,*ndh*G,*ndh*H,*ndh*J,*ndh*K
	Subunits of cytochrome b/f complex	*pet*A,*pet*B,*pet*D,*pet*G,*pet*L,*pet*N
	Subunits of ATP synthase	*atp*A,*atp*B,*atp*E,*atp*F,*atp*H,*atp*I
	Large subunit of rubisco	*rbc*L
	Subunits photochlorophyllide reductase	*chl*B,*chl*L,*chl*N
Self-replication	Proteins of large ribosomal subunit	*rpl*14,*rpl*16,*rpl*2,*rpl*20,*rpl*22,*rpl*23,*rpl*32,*rpl*33,*rpl*36
	Proteins of small ribosomal subunit	*rps*11,*rps*12,*rps*14,*rps*15,*rps*18,*rps*19,*rps*2,*rps*3,*rps*4,*rps*7,*rps*8
	Subunits of RNA polymerase	*rpo*A,*rpo*B,*rpo*C1,*rpo*C2
	Ribosomal RNAs	*rrn*16,*rrn*23,*rrn*4.5,*rrn*5
	Transfer RNAs	*trn*A-UGC,*trn*C-GCA,*trn*D-GUC,*trn*E-UUC,*trn*F-GAA,*trn*G-GCC,*trn*G-UCC,*trn*H-GUG,*trn*I-CAU,*trn*I-GAU,*trn*K-UUU,*trn*L-CAA,*trn*L-UAA,*trn*L-UAG,*trn*M-CAU,*trn*N-GUU,*trn*P-GGG,*trn*P-UGG,*trn*Q-UUG,*trn*R-ACG,*trn*R-CCG,*trn*R-UCU,*trn*S-GCU,*trn*S-GGA,*trn*S-UGA,*trn*T-GGU,*trn*T-UGU,*trn*V-GAC,*trn*V-UAC,*trn*W-CCA,*trn*Y-GUA,*trnf*M-CAU,*trn*I-CAU,*trn*S-GCU
Other genes	Maturase	*mat*K
	Protease	*clp*P
	Envelope membrane protein	*cem*A
	Acetyl-CoA carboxylase	*acc*D
	c-type cytochrome synthesis gene	*ccs*A
	Translation initiation factor	*inf*A
Genes of unknown function	Conserved hypothetical chloroplast ORF	*ycf*1,*ycf*12,*ycf*2,*ycf*3,*ycf*4,*ycf*68,*ycf*12
