# Supplementary material for: The complete chloroplast genome of Keteleeria evelyniana Mast var. pendula Hsüeh (Pinaceae), a species with extremely small populations in China
Source: Mitochondrial DNA B Resour. 2024 Apr 26;9(4):557–62. doi: 10.1080/23802359.2024.2345780 (PMC11057466; doi:10.1080/23802359.2024.2345780)
Supplement: Supplemental Material [file TMDN_A_2345780_SM4356.pdf]

Supplemental Table 1 Materials utilized in the study

Sample ID	Species	Collection	Identifier	Status	Accession number
1	*Keteleeria evelyniana* var. *pendula*	NCBI	Yang, G.	Fresh leaves	ON756024
2	*Keteleeria fortunei*	NCBI	Li, Y.	Fresh leaves	MN165037
3	*Keteleeria davidiana*	NCBI	Zhang, M.	Fresh leaves	MW580774
4	*Keteleeria evelyniana*	NCBI	Peng, J.	Fresh leaves	MW043479
5	*Ables alba*	NCBI	Li, G. Y.	Fresh leaves	MH706724
6	*Ables balsamea*	NCBI	Shao, Y.	Fresh leaves	MH706725
7	*Pseudorix amabilis*	NCBI	Sudianto, E.	Fresh leaves	NC030631
8	*Pinus densiflara*	NCBI	Kim, S. C.	Fresh leaves	MF990371
9	*Pinus yunnanensis*	NCBI	Li, M.	Fresh leaves	MK007968
10	*Pinus lambertiana*	NCBI	Cronn, R.	Fresh leaves	NC011156
11	*Pinus koraiensis*	NCBI	Noh,E. W.	Fresh leaves	NC004677
12	*Pinus gerardiana*	NCBI	Cronn,R. C.	Fresh leaves	NC011154
13	*Pinus nelsonii*	NCBI	Cronn,R. C.	Fresh leaves	NC011159
14	*Pinus monophylla*	NCBI	Cronn,R. C.	Fresh leaves	NC011158
15	*Pinus contorta*	NCBI	Cronn,R. C.	Fresh leaves	NC011153
16	*Pinus taeda*	NCBI	Liu,T. Y.	Fresh leaves	NC021440
17	*Pinus thunbergii*	NCBI	Wakasugi, T.	Fresh leaves	NC001631
18	*Pinus massoniana*	NCBI	Huang,S. W.	Fresh leaves	NC021439
19	*Picea abies*	NCBI	Nystedt, B.	Fresh leaves	HF937082
20	*Picea glauca*	NCBI	Jackman,S.D.	Fresh leaves	KT634228
21	*Picea sitchensis*	NCBI	Cronn,R. C.	Fresh leaves	NC011152
22	*Cathaya argyophylla*	NCBI	Lin, C.	Fresh leaves	AB547400
